# Toward a standardized quantitative and qualitative insect monitoring scheme

**DOI:** 10.1002/ece3.6166

**Published:** 2020-04-02

**Authors:** Axel Hausmann, Andreas H. Segerer, Thomas Greifenstein, Johannes Knubben, Jerôme Morinière, Vedran Bozicevic, Dieter Doczkal, Armin Günter, Werner Ulrich, Jan Christian Habel

**Affiliations:** ^1^ Bavarian Natural History Collections Munich Germany; ^2^ HIPP Pfaffenhofen (Ilm) Germany; ^3^ Advanced Identification Methods GmbH (AIM) Munich Germany; ^4^ Department of Ecology and Biogeography Nicolaus Copernicus University Torun Toruń Poland; ^5^ Evolutionary Zoology Department of Biosciences University of Salzburg Salzburg Austria

**Keywords:** biomass, community composition, DNA metabarcoding, insect decline, light trapping, Malaise trap, monitoring, Red List species, species richness

## Abstract

The number of insect species and insect abundances decreased severely during the past decades over major parts of Central Europe. Previous studies documented declines of species richness, abundances, shifts in species composition, and decreasing biomass of flying insects. In this study, we present a standardized approach to quantitatively and qualitatively assess insect diversity, biomass, and the abundance of taxa, in parallel. We applied two methods: Malaise traps, and automated and active light trapping. Sampling was conducted from April to October 2018 in southern Germany, at four sites representing conventional and organic farming. Bulk samples obtained from Malaise traps were further analyzed using DNA metabarcoding. Larger moths (Macroheterocera) collected with light trapping were further classified according to their degree of endangerment. Our methods provide valuable quantitative and qualitative data. Our results indicate more biomass and higher species richness, as well as twice the number of Red List lepidopterans in organic farmland than in conventional farmland. This combination of sampling methods with subsequent DNA metabarcoding and assignments of individuals according depending on ecological characteristics and the degree of endangerment allows to evaluate the status of landscapes and represents a suitable setup for large‐scale long‐term insect monitoring across Central Europe, and elsewhere.

## Graphical abstract

Malaise trap on the grassland of organic farmland.

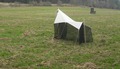

## 1 INTRODUCTION

There is a significant decrease in insect diversity and insect abundances across major parts of Central Europe for the past decades (Conrad, Woiwod, Parsons, Fox, & Warren, 2004; Fox, 2013; Hallmann, Foppen, Turnhout, Kroon, & Jongejans, 2014; Maes & van Dyck, 2001; Thomas et al., 2004; Thomas, 2005). Studies document a decrease in species richness, species abundances (Wenzel, Schmitt, Weitzel, & Seitz, 2006), and biomass (Hallmann et al., 2017) and shifts in species composition (Habel et al., 2016). These negative trends have been detectable since the 1950s, while major losses occurred during the past two decades (Habel et al., 2016). Hereby, species with specific habitat requirements (e.g., species demanding specific food plants or habitat structures) decreased particularly during the past years (Sodhi, Brook, & Bradshaw, 2009). But, also local populations and thus densities and abundances of rather generalist species, using a large variety of resources, decreased significantly (Sodhi et al., 2009). This trend was exemplary shown by the 75% reduction of biomass from flying insects over past three decades (Hallmann et al., 2017).

Previous studies on insect decline mainly focused on one single proxy, for example species richness, species abundance, species composition, or biomass (Sanders & Hess, 2019), but rarely considered all parameters in parallel. Furthermore, most existing studies on insect decline show various shortcomings (Saunders, 2017). (a) Studies refer to single species only, and thus, the validity of general trends is questionable (Reichholf, 2005, 2006, 2008); (b) studies only consider few time steps (Augenstein, Ulrich, & Habel, 2012; Filz, Engler, Stoffels, Weitzel, & Schmitt, 2013; Hallmann et al., 2017; Wenzel et al., 2006) and thus obtained changes in species richness, and community composition might be not representative for a larger time period; (c) data collected mostly refer to a geographically restricted area (Habel et al., 2016) cannot be translated to other regions; and (d) most long‐term data sets are of low quality, with multiple data gaps (Desender, Dekoninck, Dufrêne, & Maes, 2010). Most studies present more than one of these limitations (McGill, Dornelas, Gotelli, & Magurran, 2015). Thus, there is an urgent need to set a standardized insect monitoring scheme across Central Europe, applying consistent techniques and sampling protocols.

In this study, we combine quantitative and qualitative approaches to study trends of (a) insect diversity, (b) insect abundances, (c) insect community assembly, and (d) insect biomass. Insects were collected with Malaise traps, in combination with automated and active light trapping, at identical sites. Subsequently, we weighted the biomass and used DNA metabarcoding to calculate species richness. Catches of the light trapping were further analyzed in respect of species abundances and community composition, considering species‐specific traits. With our data collected during this first year of monitoring, we will highlight the following questions:

1. Are the methods selected suitable to assess quantitative and qualitative parameters?
2. Do data obtained from the two approaches provide congruent or diverging trends?

## 2 MATERIAL AND METHODS

### 2.1 Study area

Our study area is located in southern Germany, 15 km distant to the city Pfaffenhofen. We established four study sites, two in organic, and two in conventional farmland. Malaise traps and light traps were installed at extensively used grasslands. Both grasslands border north–south forest fringes and were mowed twice a year (June 24 and October 07 in organic farmland; and June 01 and September 07 in conventional farmland) and without any application of pesticides, however with the use of organic fertilizers. The organic farmland was cultivated without any pesticides since 64 years, but with organic fertilizer (on February 05). Artificial fertilizers were used on the conventional farmland (in 2018 on February 14 and April 05). The following pesticides were applied on the bordering rye and cornfields in conventional farmland: Broadway (130 g 0.5 L/ha; 14.4.2018), Gardo Gold (3 L/ha; 27.5.2018), Callisto (0.75 L/ha; 27.5.2018), and the shortcut Chlormequat (0.3 L/ha; 12.5.2018). Distance between organic and conventional grasslands was about 700 m.

### 2.2 Malaise traps

We installed four standard Malaise traps (B&S Entomological services), with two in each of the two farmland types (organic farmland 48.5073°N/11.5397°E (M1), 48.5072°N/11.5401°E (M2), conventional farmland 48.5090°N/11.5314°E (M3), and 48.5091°N/11.5318°E (M4)). All four Malaise traps were activated from 10 April to 19 October 2018. At each farmland type, one trap was positioned at the edge (forest fringe) and the second at 15 m distance in the open grassland. All traps were simultaneously opened and southwards positioned, with similar exposure to wind. We changed the 600‐ml sampling bottles filled with 80% ethanol simultaneously for all Malaise traps, eleven times throughout the year (April 25, May 21, June 2, 14, 26, July 9, 18, 31, August 18, September 12, October 19).

### 2.3 Light traps

Automated light traps (nonlethal traps without killing agent and a black light bulb) were positioned from sunset to sunrise, 14 times simultaneously in parallel at each of the two grassland sites, from April 10 till October 19 (14.4, 25.4, 4.5, 11.5, 21.5, 15.6, 29.6, 19.7, 26.7, 31.7, 3.8, 23.8, 15.9, 19.10.). In addition, we collected nocturnal moths actively at light towers equipped with 12V bulbs, 13 times simultaneously at each of the two grassland sites, from sunset to 01:00 am (17.4, 27.4, 5.5, 25.5, 1.6, 8.6, 16.6, 13.7, 27.7, 6.8, 16.8, 6.9, 12.10). As killing agent, we used ethyl acetate. Collected vouchers were identified by A. Hausmann and A. H. Segerer, and, in a few, difficult cases, with DNA barcoding (Canadian Centre of DNA Barcoding, CCDB).

### 2.4 Biomass

Dry and wet biomass material was weighted and analyzed separately, according to Ssymanck et al. (2018). Biomasses of macrolepidopterans and orthopterans have been separated and weighted before, and were subsequently added to the total weight. Based on the standardized methodology of Sorg, Schwan, Stenmans, and Müller (2013), species were dried according to size selection using a sieve (6.5 mm) in diameter in a 70°C oven over night (or at least for 8 hr).

### 2.5 Metabarcoding

Species identification of organic material in the Malaise traps was performed using DNA metabarcoding. However, macrolepidopterans and orthopterans were a priori sorted out of the Malaise traps and identified by experts at the ZSM. Some macrolepidopterans or fragments of them remained in the bulk sample and were subsequently detected by the metabarcoding approach. All microlepidopterans were identified with the metabarcoding approach in the bulk sample. Each single dried sample (altogether 4 × 11 = 44 samples) was homogenized in a FastPrep96 machine (MP Biomedicals) using sterile steal beads in order to generate a homogeneous mixture of arthropods and submitted for subsequent metabarcoding (conducted by AIM GmbH). Prior to DNA extraction, 1 mg of each homogenizate was weighed into sample vials and processed using adapted volumes of lysis buffer with the DNeasy 96 Blood & Tissue Kit (Qiagen) following the manufacturer's instructions. For amplification of the CO1‐5P target region and preparation of the MiSeq libraries, a 2‐step PCR was performed. First, a 313 bp long mini‐barcode region was amplified by PCR (Leray et al., 2013; Morinière et al., 2016), using forward and reverse HTS primers, equipped with complementary sites for the Illumina sequencing tails. In a subsequent PCR reaction, index primers with unique i5 and i7 inline tags and sequencing tails were used for amplification of indexed amplicons. Afterward, equimolar amplicon pools were created and size selected using preparative gel electrophoresis. Cleanup and concentration of amplicons were performed using the GeneJet Extraction Kit (Life Technologies). A bioanalyzer (High Sensitivity DNA Kit, Agilent Technologies) was used for a final check of the bp distribution and concentration of the amplicons before the creation of the final library. High‐throughput sequencing (HTS) was performed on an Illumina MiSeq using v2 (2*250 bp, 500 cycles, maximum of 20mio reads) chemistry (Illumina).

The bioinformatics processing of raw FASTQ files from Illumina was carried out using the VSEARCH suite v2.9.1 (Rognes, Flouri, Nichols, Quince, & Mahé, 2016) and Cutadapt v1.18 (Martin, 2011). Forward and reverse reads in each sample were merged using the VSEARCH program “*fastq_mergepairs*” with a minimum overlap of 10 bp, yielding approximately 313 bp sequences. Forward and reverse primers were removed with Cutadapt, using the “*discard_untrimmed*” option to discard sequences for which primers were not reliably detected at ≥90% identity. Quality filtering was done with the “*fastq_filter*” in VSEARCH, keeping sequences with zero expected errors (“*fastq_maxee*” 1). Sequences were dereplicated with “*derep_fulllength,*” first at the sample level, and then concatenated into one FASTA file, which was subsequently dereplicated. Chimeric sequences were filtered out from the FASTA file using the VSEARCH program “*uchime_denovo.*” The remaining sequences were then clustered into OTUs at 97% identity with “*cluster_size,*” a greedy centroid‐based clustering program. OTUs were blasted against a custom Animalia database downloaded from BOLD on 28 November 2018, including taxonomy and BIN information, by means of Geneious (v.10.2.5—Biomatters, Auckland—New Zealand), and following methods described in Morinière et al. (2016). This local sequence database consists of the compiled data which are based on the DNA library with more than 23,000 barcoded German animal species assembled in two major DNA barcoding campaigns: “Barcoding Fauna Bavarica” (BFB, http://www.faunabavarica.de, Haszprunar, 2009) and “German Barcode of Life” project (GBOL, http://www.bolgermany.de, Geiger et al., 2010), with nearly 250,000 vouchers curated at the Zoological State Collection Munich, Germany (http://www.barcoding-zsm.de). Data releases have been published for all major arthropod groups, that is, Coleoptera (Hendrich et al., 2015; Raupach, Hannig, Moriniere, & Hendrich, 2016; Raupach, Hannig, Morinière, & Hendrich, 2018; Rulik et al., 2017), Diptera (Morinière et al., 2019), Ephemeroptera, Plecoptera, and Trichoptera (Morinière et al., 2017), Heteroptera (Havemann et al., 2018; Raupach et al., 2014), Hymenoptera (Schmid‐Egger et al., 2019; Schmidt, Schmid‐Egger, Morinière, Haszprunar, & Hebert, 2015; Schmidt et al., 2017), Lepidoptera (Hausmann, Haszprunar, & Hebert, 2011a; Hausmann, Haszprunar, Segerer, et al., 2011), Neuroptera (Morinière et al., 2014), Orthoptera (Hawlitschek et al., 2018), Araneae and Opiliones (Astrin et al., 2016), and Myriapoda (Spelda, Reip, Oliveira Biener, & Melzer, 2011; Wesener et al., 2015).

The resulting csv file which included the OTU ID, BOLD Process ID, BIN, Hit‐%‐ID value (percentage of overlap similarity (identical basepairs) of an OTU query sequence with its closest counterpart in the database), length of the top BLAST hit sequence, phylum, class, order, family, genus, and species information for each detected OTU was exported from Geneious and combined with the OTU table generated by the bioinformatic pipeline. The combined results table was then filtered by Hit‐%‐ID value and total read numbers per OTU. All entries with identifications below 97% and total read numbers below 0.01% of the summed reads per sample were removed from the analysis. OTUs were then assigned to the respective BIN. Additionally, the API provided by BOLD was used to retrieve BIN species and BIN countries for every OTU, and the Hit‐%‐IDs were aggregated over OTUs that found a hit in the same BIN and shown in the corresponding column as % range (Table S2). To validate the BOLD BLAST results, a separate BLAST search was carried out in Geneious (using the same parameters) against a local copy of the NCBI nucleotide database downloaded from ftp://ftp.ncbi.nlm.nih.gov/blast/db/ (see Table “BIN sharing and countries” in Table S2) on 28 November 2018. Interactive Krona charts were produced from the taxonomic information using KronaTools v1.3 (Ondov, Bergman, & Phillippy, 2011) (cf. Figures [Link], [Link], [Link], [Link]).

Species identification in the Malaise trap samples was based on high‐throughput sequencing (HTS) data grouped to genetic clusters (OTUs), blasted, and assigned to barcode index numbers (“BINs”: Ratnasingham & Hebert, 2013) which are considered to be a good proxy for species numbers (Hausmann et al., 2013; Ratnasingham & Hebert, 2013). In our case, the detailed analysis of the Lepidoptera data revealed that the frequency of “false positives” (0.5%) and BIN‐sharing (1.5%) obstructing species discrimination (but nevertheless still pointing to species complexes) played a negligible role (see results for details).

### 2.6 Species composition and phenologies

We used two approaches to infer differences in species composition between organic and conventional farming sites. First, we used a random sampling model and calculated for all taxa having at least 15 OTUs the probability of having *k* joint OTUs, given that the organic farming sites had *l* and the conventional farming sites *m* OTUs, while the total number (the local pool size) was assumed as *n* = *l* + *m* *−* *k*. This probability is given by (Connor & Simberloff, 1978):

(1) $$p\left(k|n;\;l;\;m\right)=\frac{\left(\begin{array}{c} n\\ k \end{array}\right)\left(\begin{array}{c} n-k\\ m-k \end{array}\right)\left(\begin{array}{c} n-m\\ l-k \end{array}\right)}{\left(\begin{array}{c} n\\ m \end{array}\right)\left(\begin{array}{c} n\\ l \end{array}\right)}$$

and has the random expectation of *k*_exp_ *=* *lm*/*n* OTUs. Significant differences ∆*k* *=* *k*_exp_ − *k* point to differences in community composition. We note that the observed probabilities *p* strongly depend on the pool size *n* and cannot be compared among taxa directly. Therefore, we also estimated from Equation (1) the required number of OTUs, *n*_req_ necessary to obtain the observed *k* at the 5% error level. From *n*_req_, we also obtained the (minimal) degree of undersampling = 100 (1 − *n/n*_req_).

We calculated Spearman's rank‐order correlations (*r_S_*) between all species, which jointly occur at both farming sites. Significantly, negative *r_S_* values indicate structural differences between the two communities in terms of relative abundances. We used one‐way ANOVA to infer the difference in extinction probabilities between both farming types using average OTU abundance at both sites as the dependent variable.

To infer whether organic and conventional farming influence the phenology of arthropods, we first analyzed the combined phenologies of six major arthropod taxa (Araneae, Coleoptera, Diptera, Hemiptera, Hymenoptera, and Lepidoptera). We then assessed for each species whether the peak of emergence was identical in time (within the same sample period) between and within each farming type (OG˄IG, OF˄IF, OG˄OF, and IG˄IF). Counts of the numbers of these joint emergences in comparison with the total numbers of joint occurrences indicate similar or divergence (habitat specific) phenology.

## 3 RESULTS

### 3.1 Biomass

Weight of wet and dry biomass significantly correlated across all samples (Table 1, Table S1). Malaise traps set in the organic farmland (M1–M2) yielded 2.7 times higher amount of wet biomass (i.e., 2.6 times based on dry biomass) compared with the traps set in conventional farmland (M3–M4). The open grassland trap of the organic farming (M1 versus M3) revealed 1.5 times more biomass (i.e., 1.4 times based on dry biomass) and the forest fringe trap (M2 versus M4) 3.8 times more (i.e., 3.6 times based on dry biomass).

**Table 1:** Biomass of Arthropoda and Euarthropoda (in g) obtained from Malaise traps (wet and dry)

	Organic M1	Organic M2	Convent. M3	Convent. M4	Organic M1 + M2	Convent. M3 + M4	Total M1‐M4
Biomass wet	786.4	2,334.7	530.2	619.7	3,121.1	1,149.9	4,271.0
Biomass dry	114.4	356.5	84.2	97.4	470.9	181.6	652.5
Quotient wet/dry	6.9	6.5	6.3	6.4	6.6	6.3	6.5

Note

M1–M2: organic farming (organic); M3–M4: conventional farming (convent.). Detailed sampling sites are provided in Table S1.

### 3.2 Metabarcoding and BIN discovery

The high‐throughput sequencing (HTS) of the samples collected with four Malaise traps yielded a total of 3,117,887 sequences (paired‐end) for Arthropoda and (a few) Euarthropoda (6,235,774 sequences in total), with good sequence quality scores (FastQC). After quality filtering, 2,215,879 sequences were kept, and after dereplication and clustering, 18,531 genetic clusters were obtained, with 6,321 OTUs remaining after chimera detection. Out of these, 6,316 found BLAST hits in the BOLD database. Entries with sequence identity below 97% and total read numbers below 0.01% of the summed reads per sample were removed from further analysis. The remaining OTUs (4,506) could be assigned to 3,890 barcode index numbers (BINs) which are considered a good proxy for species numbers (see our discussion). Best represented orders were Diptera with 1,867 BINs, Hymenoptera (1,083), Lepidoptera (412), and Coleoptera (254) (see Table 2). In total, single traps collected the following numbers of species: trap M1 yielded 1,910 BINs, M2 2,776 BINs, M3 1,740 BINs, and M4 2,143 BINs. Comprehensive lists of BIN numbers, blasted species names for all Malaise trap samples, are provided in Table S2. Interactive KRONA files showing details on the BLAST results for the four sites are accessible in the Figure S1.

**Table 2:** BIN numbers (barcode index numbers, i.e., number of genetic clusters, equivalent with species numbers) of Arthropoda and Euarthropoda analyzed from biomass collected Malaise traps

	Organic M1	Organic M2	Convent. M3	Convent. M4	Organic M1 + M2	Convent. M3 + M4	Total M1–M4
Diptera	977	1,473	901	1,137	1,654	1,373	1,867
Hymenoptera	482	668	461	594	849	781	1,083
Lepidoptera	174	315	136	175	364	228	412
Coleoptera	114	168	91	103	212	149	254
Others	163	152	151	134	217	195	274
Total BINs (Arthropoda s.l.)	1,910	2,776	1,740	2,143	3,294	2,726	3,890

Note

M1–M2: organic farming (organic); M3‐M4: conventional farming (convent.). A detailed list of species and BIN numbers are provided in Table S2.

The Malaise traps on the organic farm (M1–M2) yielded 21% more species (BINs) than those of the sites under conventional farming regime (M3–M4). The open grassland trap of the organic farming (M1 versus M3) revealed 10% more species (BINs), the forest fringe trap (M2 versus M4) 30% more. The strongest divergences are found in the holometabolic orders Coleoptera (+43% on the organic farm) and Lepidoptera (+59%), of which the majority of species have ectophagous, phytophagous larvae.

The Lepidoptera fraction of the Malaise trap samples was studied in detail (merging BIN splits, separating BIN sharers, ruling out false positives, and checking plausibility of data) revealing a total of 412 recorded species (Table 3). For a detailed list of species, see Table S3. The set of 412 species included 424 BINs, of which 12 (3.6%) referred to multiple but conspecific genetic clusters (additional haplotypes). Five cases of BIN‐sharing species could be found; in three cases (0.7%), intra‐BIN divergences allowed for species discrimination (*Plusia stenochrysis* rather than *P. chrysitis*; *Mesapamea secalis* rather than *M. secalella, Conistra vaccinii* rather than *C. ligula*); in two additional cases (0.5%) (*Scoparia ambigualis/S. basistrigalis* and *Yponomeuta padella/Y. cagnagella*), this was not possible because of identity or overlap of nonconspecific sequences. The occurrence of both *Scoparia ambigualis* and *S. basistrigalis* in the HTS sequences, however, was ascertained by their phenology (*S. ambigualis*: May–June; *S. basistrigalis*: mid‐July and late July). Two false positives (2/412 = 0.5%) were ruled out among the Lepidoptera data, one referring to a Neotropical skipper (*Bolla atahuallpai*), which was deposited with an erroneous *Wolbachia* gene sequence on GenBank, and the other to a Neotropical nymphalid, *Agraulis vanillae*, which got a 98% and 99% match in BOLD and GenBank blasting, but which had only 9 reads in the Malaise trap M2 most likely due to lab contamination. According to the cleaned data, the Malaise traps on the organic farm (M1–M2) yielded 364 lepidopteran species, 136 more (+59%) than the Malaise traps on the grassland under conventional farming regime (228).

**Table 3:** Species number of Lepidoptera collected with Malaise traps, after taxonomic data validation (merging BIN splits, separating BIN sharers, ruling out false positives)

	Organic M1	Organic M2	Convent. M3	Convent. M4	Organic M1 + M2	Convent M3 + M4	Total M1‐M4
Butterflies (Rhopalocera)	13	14	6	8	17	10	18
Larger moths (Macroheterocera)	64	150	50	73	165	96	187
Micromoths (Microlepidoptera)	97	151	80	94	182	122	210
Total number (Lepidoptera)	174	315	136	175	364	228	414

Note

M1–M2: organic farming (organic); M3–M4: conventional farming (convent.). A detailed list of species is given in Table S3.

### 3.3 Species richness and abundance of larger moths at light and in malaise traps

Species richness and abundance of larger moths (Macroheterocera) from light traps confirm the results and trends from Malaise traps: Light trapping at the two sites between 10 April and 19 October 2018 yielded in total 2,639 macroheteroceran individuals representing 286 species. A detailed list of all Lepidoptera species collected at light is given in Table S4. Light trapping in organic farmland yielded 256 macroheteroceran species, 34 more (+15%) if compared with the species numbers collected in conventional farmland (222 species; cf. Table 4). Also, abundance was higher, with 1,957 individuals at the location in organic farmland, 679 more (53%) in comparison with the sampling sites set in conventional farmland (1,278 individuals). In the Malaise traps, we found 725 macrolepidopteran specimens (209 in M1, 380 in M2, 51 in M3 and 85 in M4), organic farmland yielded 4.3 times more macrolepidopteran specimens (M1–M2: 589 specimens) if compared with conventional farmland (M3–M4: 136 specimens). When combining species richness assessed with light traps and Malaise traps, we found 114 more lepidopteran species (+27%) in organic farmland, if compared with our data collected at conventional farmland.

**Table 4:** Species numbers of Lepidoptera species collected with light traps and Malaise traps

	Organic farming	Conventional farming	Total
Light trap only	169	191	190
Malaise trap only	218	138	233
Light trap and Malaise trap	146	90	181
Total species (Lepidoptera)	533	419	604

Note

For a detailed list of species see Tables S3 and S4.

### 3.4 Community composition with respect to OTUs

**Figure 2:**
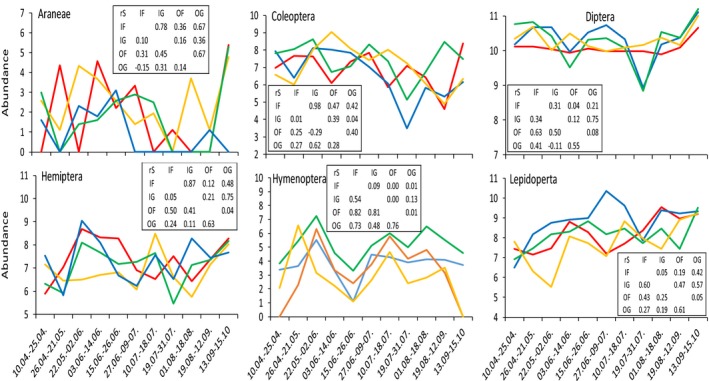
Phenologies of the four major represented insect orders represented in the organic grassland (OG: blue), organic forest fringe (OF: green), conventional grassland (IG: red), and the conventional forest fringe (IF) red). Given are ln‐transformed numbers of OTU reads per bin. The inlets provide Spearman rank‐order correlation matrices, where the lower triangle denotes correlation coefficients and the upper triangle permutation‐based significance levels

For all sufficiently rich taxa, except for Araneae and Orthoptera, we found significantly lower numbers of joint OTUs than what would be expected from a random sample model, assuming complete sampling of the local species pool (Table 5). However, even a small degree of undersampling (<10%; Table 5) was in accordance with the observed degree of joint species. These results were corroborated by the high and significant rank correlations between the jointly occurring species at both study sites indicating highly similar community composition and abundance distributions between the common farmland species (Table 5). The proportions of species occurring only in the organic farmland were higher than those in the conventional farmlands except of the species‐poor taxa Araneae, Orthoptera, and Psocoptera (Figure 2). One‐way ANOVA showed that the absence of an OTU was highly significantly linked to its overall abundance. Rare OTUs were most prone to becoming extinct in one of the farms (*F* (1, 3,892) = 89.01, *p* < .001).

**Table 5:** Results of the random sample model for expected and observed numbers of OTUs that were found at both study sites

Taxon	Total OTU	Organic farming OTU	Conventional farming OTU	Joint OTU	Expected joint OTU	Total OTU required for observed overlap	Percentage undersampling	Spearman's *r*
Araneae	23	12	20	9	10	24	4.2	.52
Coleoptera	254	213	149	108***	124	274	7.3	.31***
Diptera	1,867	1,654	1,373	1,160***	1,216	1,946	4.1	.67***
Hemiptera	174	143	128	97***	105	180	3.3	.51***
Hymenoptera	1,083	848	782	547***	612	1,195	9.4	.58***
Lepidoptera	380	335	201	156***	177	413	8.0	.47***
Orthoptera	20	17	18	15	15	20	0.0	.66**
Psocodea	19	12	12	5	8	20	5.0	.2

Note

Also given are the total numbers of OTUs to obtain the observed number of joint occurrences at the 5% error level. Spearman's *r* provides the rank‐order correlation in abundances of species jointly occurring between organic and conventional farming sites.

** *p*(*r* = 0) < .01.

*** *p*(*r* = 0) < .001.

### 3.5 Species communities

Altogether, 47 species of the Bavarian “Red Book of threatened Lepidoptera species” (Bayerisches Landesamt für Umweltschutz, LFU, 2003; 2005) were found in the Malaise traps and at light (Tables S3 and S4). Forty species of the Bavarian “Red Book” were recorded on the organic farm (16 at light, 26 in Malaise traps, two overlapping in both methods) and 21 only on the farm under conventional farming regime (nine at light, 16 in Malaise traps, four overlaps).

**Figure 1:**
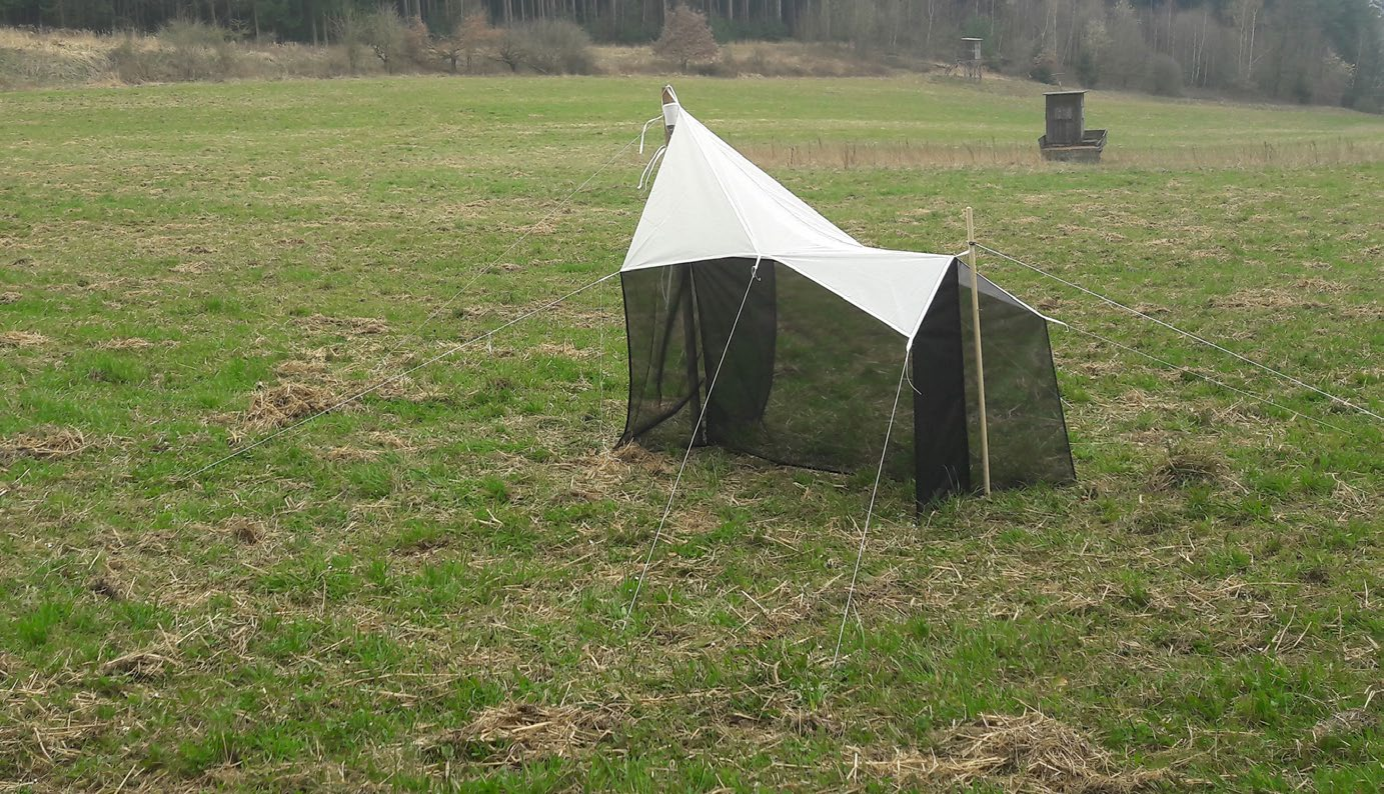
Malaise trap on the grassland of the organic farmland

Only three out of 36 Spearman rank‐order correlations for major arthropod taxa between abundances across both farming types and habitats (grassland, forest fringe) were negative (Figure 1). The average Spearman correlations within each farming type (grassland–fringe) were *r*_S_ = .38 ± .08 and between the farmland types *r*_S_ = .49 ± .07. The respective average correlation for the organic grassland–conventional grassland combination was *r*_S_ = .31 ± .08 and for the organic forest fringe–conventional forest fringe combination *r*_S_ = .20 ± .08. The respective average organic grassland–organic fringe correlation was *r*_S_ = .20 ± .09, and the conventional grassland–conventional fringe correlation was *r*_S_ = .11 ± .10 indicating that grasslands and forest fringes have similar average phenologies irrespective of farming type (Figure 1).

The species‐specific analysis showed that more than 50% (in most comparisons more than 65%) of species had identical peaks of emergence across the habitat types (Table 6). Both grasslands had less similar species emergence peaks than both forest fringes (Table 1). Importantly, organic grasslands and forest fringes were more similar in species phenology than the intensively managed grasslands and fringes (Table 6).

**Table 6:** Numbers of OTUs for six large arthropod taxa and for all taxa that occurred jointly in pairs of habitats (organic grasslands *OF*, organic forest fringes *OF*, intensively managed grasslands *IF*, intensively managed forest fringes *IF*, and the numbers and percentages of species that had temporary identical peaks

Taxon	Joined occurrences		Identical peaks		Percentage identical peaks		Joined occurrences		Identical peaks		Percentage identical peaks	
	OG˄IG	OF˄IF	OG˄IG	OF˄IF	OG˄IG	OF˄IF	OG˄OF	IG˄IF	OG˄OF	IG˄IF	OG˄OF	IG˄IF
Araneae	3	6	2	4	66.7	66.7	4	3	2	1	50.0	33.3
Coleoptera	52	68	37	51	71.2	75.0	71	46	51	24	71.8	52.2
Diptera	652	984	469	764	71.9	77.6	846	706	663	452	78.4	64.0
Hemiptera	73	68	51	48	69.9	70.6	77	65	60	42	77.9	64.6
Hymenoptera	335	447	202	306	60.3	68.5	377	338	251	196	66.6	58.0
Lepidoptera	79	116	53	87	67.1	75.0	115	74	88	49	76.5	66.2
All arthropods	1,103	1,606	752	1,199	68.2	74.7	1,395	1,156	1,035	710	74.2	61.4

## 4 DISCUSSION

Our approach combines quantitative and qualitative assessments and data. Our methods include the calculation of biomass with the use of high‐throughput sequencing of the biomass collected with Malaise traps to calculate species richness. We complement these quantitative data with a qualitative insect assessment, using light traps. We applied automated light traps to collect nocturnal lepidopterans and performed active sampling of nocturnal lepidopterans on light towers. These data provide information on species abundances and community composition. We completed this last step by assigning ecological traits to each single species, such as Red List status. Our data show parallel temporal fluctuations and show higher diversities and abundances, more biomass, and a much larger number of Red List nocturnal lepidopterans in organic farmland if compared with the samples collected in conventional farmland.

### 4.1 Complementing quantitative and qualitative assessment

The combination of a quantitative and qualitative data set provides various advantages, while each single method shows different strengths, but also shortcomings. DNA metabarcoding allows analyses of large arthropod bulk samples of several thousand individuals from hundreds to thousands of species within only few weeks (Creedy, Ng, & Vogler, 2019; Morinière et al., 2016), including comprehensive species inventories (Wang, Srivathsan, Foo, Yamane, & Meier, 2018; Yu et al., 2012), species turnover (Doi et al., 2016), species composition in transects (Ji et al., 2013), seasonal fluctuations (Figure 2), and other aspects. This is a major advantage, especially when assessing insect species, which frequently occur in high densities and represent a very diverse group of organisms, with a large number of species difficult to identify, or which are still not yet described (Page, 2016; Stork, 2018). Traditional, morphology‐based, “manual” identification of such large amounts of insect samples is time‐ and cost‐consuming, and thus usually unrealistic as it is shown by a recent analysis of German malaise traps through experts requiring nine years for performing sorting and a morphology‐based identification of roughly 10% of the collected individuals (Ssymank & Doczkal, 2017). Thus, an automated, time‐, and cost‐efficient system is the prerequisite to establish a large‐scale long‐term insect monitoring. Another advantage of this technique is that time‐integrated sampling techniques with DNA metabarcoding allow parallel assessment. Synchronous sampling is in particular for insects of high relevance, as this group of organisms may frequently underlie severe seasonal fluctuations. Thus, parallel sampling with automated and standardized techniques is the prerequisite, especially when using these data for comparative study setups and questions (comparing the impact of farming intensities, Sanders & Hess, 2019). Lastly, DNA metabarcoding of bulk samples from Malaise traps considers a very large fraction of the multicellular fauna and thus provides a very strong explanatory power, if compared to other approaches, such as picking a limited number of indicator species or taxonomic groups (Ssymanck et al., 2018).

The method applied here is time and cost efficient and allows a standardized sampling with standardized molecular analyses of the material collected. Based on the approaches applied, we obtained valuable information on insect diversity and biomass. However, pure data on the number of species and the amount of biomass provide little information on the status of an ecosystem or of entire landscapes, and information on the abundance of species and species compositions is crucial (Elbrecht & Leese, 2015). Thus, additionally we performed selective sampling of a well‐known taxonomic group to assemble supplementary data to our DNA metabarcoding approach. Nocturnal lepidopterans are a suitable group to evaluate ecosystem health and the status of entire landscapes (Holloway, 1980). Nocturnal lepidopterans are very rich in species and comparatively well understood in terms of taxonomy and ecology (Haslberger & Segerer, 2016; Hering, 1951; Kristensen, 1999; Scoble, 1995). Many representatives of this taxon react highly sensitive onto environmental changes such as land use intensification and the deterioration of habitat quality (Bayerisches Landesamt für Umweltschutz, LFU, 2003; Ekroos, Heliölä, & Kuussaari, 2010; Habel, Ulrich, Biburger, Seibold, & Schmitt, 2019; Sánchez‐Bayo & Wyckhuys, 2019). Thus, this second approach, automated light trapping and active sampling at a light tower, provided in total 3,738 individuals and 371 lepidopteran species, including 20 Red List species. The use of further ecological characteristics for single species provides very relevant information on potential community shifts (e.g., reductions of evenness, decline of species with specific habitat requirements, shifts in species composition, Habel et al., 2016). Thus, this second, qualitative approach yields detailed information on species abundance and the structure and quality of a species community. In addition, with the use of light traps we obtain information from a larger, landscape scale, as this method attracts individuals from adjoining habitats, and thus, the collected samples rather reflect the surrounding area, than only from the grassland patch on which data collection was conducted (Truxa & Fiedler, 2012).

### 4.2 Organic versus conventional farming

We sampled insects in organic and conventional farmland. Our data reveal higher biomass and species richness, and twice the number of threatened nocturnal lepidopterans in organic farmland if compared with our sampling sites in conventional farmland. However, we have to interpret these findings with caution, as data collection was performed on only two sites of each farming type. Thus, we only may conclude vague trends, but cannot (yet) derive very meaningful conclusions. However, our trends are in accordance with other studies showing a significant loss of biomass and reduced species richness in conventional farmland (Sanders & Hess, 2019). While we found significant differences of arthropod biomass and species richness between the two farmland types, we found similar species community composition across all four sampling sites, which is congruent with other studies (Gibson, Pearce, Morris, Symmondson, & Memmott, 2007), but also contrasts with previous work (Boutin, Martin, & Baril, 2009; Tsutsui, Kobayashi, & Miyashita, 2018). Our data show that Red List species mainly occur in organic farmland, which goes in line with Sanderson‐Bellamy, Svensson, Brink, Gunnarsson, and Tedengren (2018), showing that specialist species (frequently also found on Red Lists) suffer in particular under agricultural intensification.

## CONFLICT OF INTEREST

TG, AG, and JK are permanent employees of the HIPP Company which operates the organic farm, one of the two study sites. The other authors declare no competing interests.

## AUTHOR CONTRIBUTIONS

Thomas Greifenstein, Armin Günter, and Dieter Doczkal conducted the field work. Axel Hausmann, Jerome Morniere, Vedran Božičević, and Werner Ulrich performed data analyses. Axel Hausmann, Jan Christian Habel, and Werner Ulrich wrote the manuscript, with further contributions by all other authors. **Axel Hausmann**: Conceptualization (equal); data curation (equal); investigation (equal); methodology (equal); project administration (equal); supervision (equal); writing‐original draft (equal); writing‐review and editing (equal). **Andreas Segerer**: Conceptualization (equal); data curation (equal); investigation (equal); methodology (equal); project administration (equal); writing‐original draft (equal); writing‐review and editing (equal). **Thomas Greifenstein**: Conceptualization (equal); data curation (equal); methodology (equal); project administration (equal); writing‐original draft (equal); writing‐review and editing (equal). **Johannes Knubben**: Funding acquisition (equal); investigation (equal); project administration (equal); writing‐original draft (equal); writing‐review and editing (equal). **Jerome Morniere**: Conceptualization (equal); data curation (equal); formal analysis (equal); methodology (equal); software (equal); visualization (equal); writing‐original draft (equal); writing‐review and editing (equal). **Vedran Božičević**: Data curation (equal); investigation (equal); methodology (equal); writing‐original draft (equal); writing‐review and editing (equal). **Dieter Doczkal**: Data curation (equal); methodology (equal); writing‐original draft (equal); writing‐review and editing (equal). **Armin Günter**: Data curation (equal); funding acquisition (equal); methodology (equal); project administration (equal); writing‐original draft (equal); writing‐review and editing (equal). **Werner Ulrich**: Conceptualization (equal); investigation (equal); methodology (equal); supervision (equal); validation (equal); visualization (equal); writing‐original draft (equal); writing‐review and editing (equal). **Jan Christian Habel**: Conceptualization (equal); methodology (equal); supervision (equal); validation (equal); visualization (equal); writing‐original draft (equal); writing‐review and editing (equal).

## Supporting information

Table S1

Table S2

Table S3

Table S4

Figure S1

Figure S2

Figure S3

Figure S4

Supplementary Material

## Data Availability

Collected vouchers (for the species collected with light traps) are deposited in the Bavarian State Collection of Zoology (ZSM) (Munich, Germany). Homogenized vouchers are collected with Malaise traps, and subsequent isolated DNA is deposited in the Bavarian State Collection of Zoology (ZSM) (Munich, Germany). All molecular data obtained from metabarcoding are provided in Table S2, as well as as interactive Krona files (Figures [Link], [Link], [Link], [Link]). All raw data used in this article are uploaded to the public repository Dryad, accessibly under https://doi.org/10.5061/dryad.mpg4f4qvw.
