# Supplementary material for: Toward a standardized quantitative and qualitative insect monitoring scheme
Source: Ecol Evol. 2020 Apr 2;10(9):4009–20. doi: 10.1002/ece3.6166 (PMC7244892; doi:10.1002/ece3.6166)
Supplement: Supplementary file 5 — Figure S1 [file ECE3-10-4009-s005.html]

Javascript must be enabled to view this page.

magnitude
magnitudeUnassigned

organic grassland

627276

29

29

29

29

29

29

118

118

118

118

2

2

2

2

2

2

625913

52
120

9
41

3

3

3

4

4

4

2

2

2

21

21

21

2

2

2

13

13

13

13

11

3

3

3

3

31

3

3

3

3

28

625605

2674
1077

1597

1597

1055

542

8692

508

152

29

29

327

327

8184

71

71

25
8108

1770

175

4

114

1494

33

4465

28

5

5

103
4

99

99

63

36

219

219

219

219

144
227

10

10

10

67

67

67

3

3

3

3

3

3

3

3865
25609

763

753

753

10

10

5

5

3

2

14

14

14

1399
11533

206

206

8

8

17

5

12

91

91

6638
6000

143

495

158

158

15

15

746

746

274

274

113

113

146
177

31

6

6

58

58

23

194

126

68

3

3

588
232

3

353

41

41

268

268

234
105

129

274
50

224

2

2

2

2

2

337

337

5

332

20

20

2479

10

10

668

668

1776

1776

25

25

69

33

11

22

36

36

613
114

4

4

3

3

454
14

440

26

26

4

4

8

707

707

707

23

23

23

123
401

8

8

4

4

31

31

4

4

14

14

3

3

5

5

122

82

6

34

31

31

19

19

4

4

33

25

8

156
411

3

3

252

1113

1108

1108

5

5

10

10

111

111

111

93

93

93

21

6

6

15

15

3019

3019
44

2975

82194
34622

2
36

2

2

32
30

2

13890

2443

2443

8984

8668

31

285

1797

2

1795

255

229

26

411

411

1007
52

8
12

4

10

10

16

3

4

9

6

6

13

13

18

7

7

4

4

103

27

11

11

10

2

42

3

3

7
14

5

2

10

7

3

3

4

112

112

218
58

160

82

77

3

2

19

19

23
47

21

3

2
169

2

130

5

14

16

2

74
55

19

2

2

2

3

3

2

2

2

2

2

276

237

14
4

7

3

25

4167

6

6

47

3

44

481

17

13

66

13

372

1468

1098

63

307

141

141

11

11

207

207

56

56

24

24

64

64

46

20

26

20

4

16

295

230

34

31

5

5

15

15

170

157

13

860

5

25

84

746

19

19

158

158

16

16

44

44

14

14

8

64

13

13

51

51

2

2

2

2
41

29

4

25

10

10

2

2

6

6

6

177

177

41

21

6

109

2

109
8741

8

8

2325

2

45

2278

89

89

429

26

403

30

30

5751

5679

19

48

5

7
4

3

3

3772

3547
3

10

431

165

31

140

420

54

1779

486

28

4

4

43

5

38

178

178

656

6

6

17

17

67

9

58

326

11

307

8

240

129

111

12
5

7

7

13420
1294

84

58

26

1146

4

4

38

38

29

29

33

13
7

6

10

10

30

19

11

2749

2749

64
22

42

24

2

7

15

2

2

10

13

13

641

641

12

4

8

3

3

670
1130

11

372

77

63

13

13

7

1126

1126

7

7

67

67

22

22

352

98

98

4

22

9

13

36

36

2

2

16

16

12

12

2

2

5

5

209

92

34

83

151

151

604

11

73

520

3

3

40

6

6

37

37

103

103

5

5

20

20

6

6

37

46

46

105

105

30

30

15

15

96

96

51

51

58

58

44

44

10

14

14

8

8

43

43

2

2

25

25

226

8

8

71
37

34

627
596

31

24

24

177
170

7

9

9

1013

188

825

29

29

75

12

12

3

3

17

17

48

48

40

40

38

38

32
52

20

12
24

7

7

5

5

224

146

3

10

32

37

16

48

60

32

5

23

18

18

9

9

9

23

12

4

8

5

5

6

6

488

27

21

6

40

40

89

25

64

9

9

43

43

280

114

166

242

242

242

48

48

6

42

10

6
10

4

6

6

6

143

119

119

24

24

67

67

67

702

26

26

26

179

179

179

479

479

479

18

18

18

113238
379732

16246

135

5

130

51

13

38

47

47

5

5

78

31

47

711

711

9

9

570

570

3

3

17

17

235

235

105

105

144

144

56

56

5

5

39

39

12

12

2057

2057

3

2

2

3161

3161

118

118

3504

3504

1529

1529

12

12

297

297

78

78

45

45

8

8

29

5

5

76

76

174

174

2

35

35

63

63

125

125

449

449

2164

6

2158

41

41

47

47

1203
36

423

13

21

386

3

5

5

112

4

58

50

274

82

6

4

3

155

24

5

5

22
95

4

61

8

82

10

24

19

13

16

148

28

71

24

22

3

23

23

15

15

15

2371

375

360

15

1996

1815

100

81

16

265

265

265

4335

4335
267

260

243

35

83

61

1844

4

78

23

16

683

227

480

2

24

5

88

88

88

4777
65

199

111

88

691

691

6

6

721

627

45

49

686

11

675

8

8

1477

1477

6

169
918

90

21

19

28

8

14

569

707

43

43

664

664

712

25

25

39

39

32

32

42

39

3

61

61

122

122

13

13

210

210

110

110

49

49

9

9

15
681

3

3

150

90

2

44

3

11

8

8

7

7

2

2

105

105

3

3

99

99

5

5

2

56

49

7

3

3

38

38

18

18

24

24

87

9

78

2

19

19

30

30

5

21088

4

4

52

52

140

140

11
9973

26

564

5682

30

6

65

2523

31

997

38

64

64

334

334

2255

827

67

220

24

759

358

28

28

1480

77

1403

11

11

218

218

33

33

245
219

24

2

126

126

5388

2328

166

198

189

2485

22

619

169

450

53

7

46

65

65

712
264

5

5

12

8

2

4

4

2

34

48

10

3

20

8

8

7

7

14

58

24

3

25

10

3

3

3

58

44

14

6

6

26

48

3

4

29

2

2

27

70

28

28

28

505

348

5

12

14

317

6

6

34

5

18

11

19

19

35

12

23

22

22

3

3

7

7

25

25

6

6

5084

5084

5084

414

8

4

4

70
89

19

133

133

171

160

11

13

4

9

191

171

18

61

92

20

20

67

49

49

18

18

16
880

498

498

366

366

379
1134

3

3

102

357

76

39

242

293

31

262

5034

26

5008
2

543

4463

2
47303

715

715

141

46430
176

1202

17173

81

477

9272

203

3194

12701

1587

3

4

146

211

15

7

8

537
4608

12
124

112

2

2

46

36

36

279
3198

10

25

171

424

2

61

404

5

20

32

82

14

7

465

91

11

45

68

151

11

6

40

37

14

473

212

32

6

25

47

3

3

52

24

11

11

8

12

5

56

56

8

317

41

87

189

8

36

19

17

51

19

16

16

1813

1813

2843

2843

1052

92

1699

349

125

125

15

15

161

48

48

25606

4
1019

113

650

30

5

217

31

31

24556

139

2105

1318

158

9692

7790

3354

1794

53

53

6

6

762

23

21

2

3

3

8

8

483

483

393

264

129

63

50

13

557

88

88

439

439

18

18

12

12

36

36

36

25202
207

3
1287

1284

649
10077

9428

235
173

62

1581

1581

3

3

15

15

68

68

5441

5441

6

6

145

145

69

69

212

212

287

287

44

44

285

285

3979

2975

824

166

14

494

494

13

13

250

70

180

144

144

360

360

3398

4

479

479

66

66

215

28

38

6

143

284

284

80

50

5

17

8

78

4

4

171

171

50

3

47

27

27

35

5

10

12

8

12
270

15

87

26

5

18

9

63

31

4

6
49

12

10

4

17

1162

3

65

3

12

6

8

460

3

143

205

213

2

24

3

12

311

269

42

113

31

82

430

197

197

233

233

9

9

9

151

137

137

14

14

1593

1593

1593

9

9

9

1277
43

426

426

54

54

56

56

104

104

594

594

26152

63

63

1319

1166

153

11

11

18987
19553

166

20

91

121

168

5

5

1892

11

7

165

1640

69

23

23

1985
2

1051

40

892

1284

1284

17

941

941

941

1955

4

4

783

549

234

1138

1138

30

30

97

97

97

1051

84

84

967

46

921

37475

32

13

19

10

10

935

2

118

50

745

20

2

2

633

633

1994

148

1846

4581
106

546

3601

328

29
13368

26

1563

11750

13

1362
314

74

82

83

809

109

30

15

64

12338

271

7965

21

53

1723

2238

67

140

91

49

1566

1548

18

392

392

973

940
93

3

229

35

60

26

473

21

33

12231

10810

6305

280

12

920

2026

17

1115

135

11

4

7

695

715

98

617

300

73

56

17

227
4

4

180

2

5

6

4

22

1681
53

307

307

38

38

755

97

591

67

55

80

80

380

380

11

2

2

8

8

8

155

40

40

40

115

111

111

4

4

118
15103

364

2

2

35

3

13

15

4

176

144

32

6

6

117

117

8

8

2

18

18

270

67

36

31

173

173

30

13

17

300

300

300

2113

1819

20

1463

205

131

2

2

267

29

8

230

25

25

330

330

3

93

234

2

2

2

559

559

559

1051

1051

1051

24
3800

34

34

84

84

24

24

236

236

2397

2397

3

3

942

942

27

27

29

29

68

68

68

78

43

43

6

6

4

4

20

20

5

5

846

846

846

9

9

9

416

3

3

75

75

97

93

4

16

16

225

212

13

3435

19

19

146

143

3

261

261

141

129

12

948

948

1900

1900

4

4

16

9

7

17

17

17

49

18

18

5

5

4

4

11

11

11

11

109

109

23

7

47

32

69

67

67

2

2

19

19

19

210

52

8

13

31

148

148

10

10

20

8

8

12

3

9

42

42

42

76

76

76

609

14

14

20

20

26

7

3

16

239

62

172

5

56

2

54

10

10

90

90

2

2

152

112

36

4

124

124

80

17

2

25

1365
108375

9311

11

11

427

427

397

397

133

133

11

11

447

259

7

181

41

35

6

4

4

167

167

26

26

719

719

6928

6928

2056

60

60

18

18

1978

1978

34

12

12

7

7

15

15

3300

542

542

1880

1880

15

15

51

51

812

812

11

11

11

58

58

58

158

3

3

15

15

140

26

36

3

22

2

51

64

35

35

29

20

9

6

6

3

3

258

258

258

9

9

9

29

29

29

306

306

306

46

42

42

4

4

57324

628

628

104

104

18

18

22301

10222

12038

41

90

90

1015

997

18

625

625

8783

8783

3330

3330

5

5

116

116

6
1821

108

245

1462

2022

2022

52

52

887

887

1771

1771

13756

13741

15

12

12

12

44

23

23

16

16

5

5

40

40

40

52

52

52

1840

1840

1840

17

17

3

14

313

313

313

54

22

22

32

32

1153

1150

1150

3

3

310

310

147

163

12

12

10

2

441

441

24

2

95

320

497

177

165

12

304

304

16

12

4

1798

149

149

261

261

3

3

93

93

1268

1268

6

6

10

10

8

4

4

15914
593

633

197

436

1323

1323

33

33

423

423

12312

12312

597

215

382

11377

634

634

6895

5194

1701

1081

710

371

216

204

7

5

426

426

65

65

386

386

28

28

16

16

122

122

188

188

882

16

835

31

66

66

38

38

3

3

139

50

89

192

192

166

12

6

6

38

3

35

116

116

40

40

40

40

6

6

6

6

117

117

20

20

85

85

12

12

1654

1654

1654

835

311

508

157

8
157

110

110

110

39

39

39

214

214

214

214

214

214

60

60

60

60

60

21

39

940

593

579

418

308

221

56

21

10

110

13

97

161

159

159

2

2

14

14

14

14

347

9

9

9

9

22

2

2

2

20

20

20

31

4

4

4

27

27

27

285

285

285

285
