# Supplementary material for: Toward a standardized quantitative and qualitative insect monitoring scheme
Source: Ecol Evol. 2020 Apr 2;10(9):4009–20. doi: 10.1002/ece3.6166 (PMC7244892; doi:10.1002/ece3.6166)
Supplement: Supplementary file 6 — Figure S2 [file ECE3-10-4009-s006.html]

Javascript must be enabled to view this page.

magnitude
magnitudeUnassigned

organic forest fringe

581436

2

2

2

2

2

2

8

8

2

2

2

2

3

3

3

3

3

3

3

3

580839

580036
51

537

203

201

193

8

2

334

334

199

4

110

18

3

47690
1714

83

4

4

48

5

3

8

27

5

31

26

5

5

5

5

31

31

31

3441
363

428

294

38

96

49

49

40

40

403

403

480

480

632

491

141

450

376

74

35

35

561

526

35

6812

31

23

8

91

91

5

5

23

6

17

12

12

1593

1593

1577

332

1245

168

168

7

7

11

11

29

29

294

155

118

21

455

365

90

4

4

4

4

17

17

37

37

97

25

8

64

91

32

59

20

20

40

40

13

13

6

6

18

18

22

9

13

38

38

816

12

45

7

25

6

327

383

11

918

760

15

143

337

337

28

16

12

10

10

89

8

5

3

54

54

27

13

14

138

68

68

45

45

25

25

366

23

23

50

12

38

94

94

53

48

5

35

35

111

111

4

4

4

3566

1201

1201

142

142

2223

2223

85

85

85

94

30

30

45

45

19

19

178

52

52

32

32

94

94

810

810

810

4

4

4

993

11

11

91

91

5

5

74

74

4

4

88

41

47

38

38

45

45

637

637

12

12

12

223

102

78

24

58

58

63

51

12

39

39

39

1582

168

168

30

30

303

303

1081

1081

15

15

15

915

276

276

49

49

4

4

25

25

55

55

47

18

29

457

457

2

2

157

41

41

48

48

9

9

59

59

43

43

15

28

84

84

61

11

9

3

86

86

11

49

7

19

2879

9

9

15

15

13

13

13

13

12

12

230

13

217

58

58

25

25

44

44

83

83

31

20

11

13

13

15

15

143

46

83

14

36

36

19

19

7

7

77

42

8

27

41

41

9

9

83

83

34

34

4

4

124

33

40

51

7

7

26

26

65

51

14

19

19

440

83

357

442

442

30

30

586

586

126

7

5

13

30

41

25

5

29

2

2

27

27

559

370

370

71

71

6

6

102

102

10

10

176

145

145

13

13

18

18

169

7

7

58

58

33

33

71

71

3693

3680

3680

13

13

342

336

196

140

6

6

31

31

31

11

2

2

9

5

4

3

3

3

18021

672

494

54

124

171

171

42

4

38

142

142

429

429

5585

5585

2068

7

2061

9

9

46

46

11

11

277

277

338

338

38

38

267

267

231

209

22

7

7

201

23

178

3542

1933

1442

167

152

152

24

24

392

392

55

34

21

384

384

150

71

79

477

477

18

18

1539

577

962

11

11

743

743

51

51

7

5

39

12

12

12

95

95

95

5

5

5

45

16

16

29

29

43

43

43

43

17731
1903

133

131

131

2

2

325
95

18

18

4

4

208

208

3

3

3

40

40

40

5

5

5

5

5

5

2425

77

77

2338

2338

2

2

8

8

102
520

3

3

92

48

44

205

5

200

5

5

72

72

5

5

18

18

15

15

3

3

402

5

5

397

358

39

9

9

9

338

109

109

229

229

926

926

926

1648

1648
51

1597

8832
454

287

238

49

3

3

32

32

78
54

24

29

29

158
675

517

70
15

55

4813
6251

1263

175

35

3

3

5

5

449

449

67

67

124

124

35

35

3

3

112
87

25

9

9

3

3

79

79

21

21

8

8

117

92

92

25

25

39
19

20

61

61

61

47
123

35

11

11

24

24

7

7

7

6

6

6

28

28

28

94

58

2

2

51

51

5

5

27

27

22

5

9

9

9

5

5

5

5

2

81338
379354

1770

136

136

149

66

83

192

102

11

79

54

54

51

2

21

4

24

7

7

19

13

6

19

19

12

12

6

6

60

16

15

29

17

17

24

24

6

6

18

18

587
55

6

190

83

20

153

10

4

24

5

28

9

25

25

7
370

5

3

16

329

3

7

2
18

16

811

247

247

555

200

273

82

9

9

89

89

89

168

168

11

14

143

180
750

34

13

13

14

10

10

9

3

6

2

2

7

7

13

13

19

408

15

3

13

8

133

236

8

8

2

2

2

2

15

15

14

14

1077

160

61

20

79

16

16

901

34

77

48

742

5466

3489

3489

14

14

726

43

683

481

469

12

6

6

750

136

614

6500

54
6495

146

117

541

50

7

216

114

1452

6

406

695

122

665

123

82

348

15

269

584

25

85

87

286

5

5

41683

37417
62

85

43

18

1123

72

19666

159

2588

7483

118

437

14

9

3557

688

487

7

79

76

633

13

9

578

3384

3384

2

2

293

4

289

34

34

34

617

565

499

66

16
52

36

60

60

60

198

151

151

47

47

61

61

23

27

11

41
7823

2007

152

35

1816

4

2838

2838

10

10

565
1336

75

4

5

3

6

82

7

48

16

6

4

485

30

129

101

28

914

914

33

33

379

379

136

52

84

14

14

14

77

9

9

51

51

17

17

1450

1450

754

359

337

1612

1612

1612

226

110

110

103

100

3

13

13

24

24

24

7798

682

4

171

507

52

52

343

205

29

53

25

6

25

153

864

5704

1277

1679

15

1064

152

833

6

3

3

672

5

5

5

43

43

43

27

27

27

16

16

1563

62

22

9

31

38

38

8

8

16

16

131

131

10

10

57

57

8

8

6

6

847

370
3

367

7

7

3

3

2
908

58

9

15

8

26

17

8

9

14
831

19

12

3

16

26

3

570

153

15

394

240

240

154

154

28
522

2

2

50

50

132

10

23

8

81

10

279

258

5

16

31

1567
177

22

354

354

75

13

58

4

202

87

115

56

5

51

8

43

20

20

162

162

431
13

61

19

13

325

5

5

12

12

401

14

14

15

15

6

6

3
10

7

10

10

209

209

78

78

21

8

13

3

3

20

20

15

15

39

468

56

50

6

34

34

7

7

5

5

180

128

52

7

7

7

5

2

172
52

48

72

933
2

68

68

85

85

82

82

9

9

370

370

37

37

273

25

248

7

22714

521
12

509

916

97

70

27

722

8

8

2443

2443

29

292

292

4

4

75

75

105

52

53

21

21

1266

1266

8

8

432

432

966

966

314

42

272

75

75

133

133

323

323

146

135

11

902

902

2263

2263

10

10

462

308

154

123

123

287

287

26

26

5970

5970

359

101

28

230

4

4

190

186

4

29

29

98

98

62

62

8

8

188

135

53

4

4

80

80

32

32

93

93

44

30

14

17

17

208

208

220

220

11

11

34

22

22

159

159

657

502

155

50

23

27

123

123

42

22

20

11

11

27

27

25

7

18

1074

20

1054

723

723

574

464

464

68

68

42

42

4213

43

4

39

11

11

110
4

53

21

32

1979

302

35

4

88

8

368

32

3

5

3

33

12

15

3

11

8

1027

6

16

410

410

252

252

184

42

142

102

83

19

8

2

6

7

7

331

2

134

8

183

4

33

474

39

374

2

44

15

4

4

170

170

95

9

2

34

21

24

5

3450
60

5

5

4

20

36

14

113

2

10

2

2

4

11

22

7

15

14

12

90

5

2

2

2

114

5

29

4

2

27

577

801

10

19

19

419

4

87

319

9

6

6

585

585

10

32

5

5

3

3

104

104

41

7

3

2

31

181

2

3

38291

570

308

262

1955

1955

580

359

4

217

7

7

61
10615

7280

39

117

7

830

43

728

122

19

148

575

646

679

337

330

2

10

28
17628

3262

12961

7

931

14

43

240

142

10
2330

241

237

5

69

1768

128

8

120

620

620

21

3

3

78

78

15

15

138

129

129

41

41

8

8

299

179

15

91

14

1456

127

49

1280

479
991

213

5

294

565

58

58

15

15

155

155

337

337

83
76

7

5
1821

5

5

12

12

20
75

9

25

14

7

20
418

316

69

13

48

20

28

801

604

4

22

133

19

5

14

404

13

9

146

6

18

176

30

6

3

3

50

3

8

3

8

28

36772

512
307

205

8

8

20824

13988

5601

257

33

214

373

358

34

34

233

233

15045
136

212

141

3

226

122

38

14167

107

107

9

9

1123
4025

52

39

13

10
1967

17

1940

16

195

195

661

533

23

105

11

11

65

27

27

6

32

7

25

63

63

63

6206

11

82

82

61
6113

326

5726

95

80

15

15

900

123

123

566

465

44

44

13

176

20

20

15

15

53

53

53

45
1259

1210

4

2

2

15293
1399

6

65

10

168

6

4235
53

4182

58

58

8

5

3

53

74

5

69

43

952
7355

17

9

130

3

188

3

113

48

8

248

36

14

45

13

9

4

45

136

113

62

7

92

1459

191

159

14

51

22

287

246

172

259

265

69

35

255

7

2

28

128

9

1402

105

3

31

71

23

3

9

513

139

17

17

11

32

2

23

296

229

60

7

13
200

4

7

176

21

37

37

2

380

12
5

7

7

93

93

118
30808

215

138

77

1932

1486

48

235

116

47

7948

7803

145

762

168

15

579

124

91

23

10

3045
195

402

2302

45

101

6583

16

5282

18

160

12

1095

7

7

107

107

52

52

82

82

434

295

9

130

184

1167

1167

3473

3064

144

124

141

16
3109

2202

891

1466

1382

9

25

41

9

8

8

6

6

6

290

290

290

613
14186

456

344

112

4

4

838

173

527

138

24

11

13

126

107

19

12

3

9

3713
132

3581

36

36

114

69

45

299

299

136

136

567

185

382

1056

1056

17

17

1681

1547

134

3

3

32

32

111

42

4

18

47

13

13

560

560

21

21

551

551

2234

2234

887

6

833

48

40

40

42

42

6
11625

43

33

37

66

34

32

229
11414

121

2088

349

225

113

162

8127

26

3
19352

287

42

97

3

14

53

78

362

296

38

28

27

27

6441

24

6417

10434
10752

49

100

142

27

3

3

231

21

210

53

53

33

33

1160

1160

26560
122

2565

7

7

164

19

143

2

101

96

5

4

4

1400

384

9

49

3

271

8

12

31

625

8

8

8

8

8

36

36

44

38

6

2

2

27

23

4

449

449

98

6

92

217

207

10

2340

21

21

36

36

42

42

127

127

104

104

225

225

20

20

2

2

9

9

79

79

119

119

1545

1545

11

11

1231

1231

1231

15

15

15

82

18

18

64

64

180

89

89

81

9

9

53

6

4

10

10

6952

6952

2316

4636

313

313

313

294

268

268

26

26

7

7

7

7

7

7

606

250

129

23

98

167

23

37

95

12

20

3

10

7

152

152

17

17

70

11

11

4

4

55

55

117
2030

84

84

8

8

14

14

773

773

779

777

2

200

200

6

4

2

32

32

7

7

10

10

89

78

51

10

9

8

11

11

25

25

25

87

87

87

24

24

24

5

5

5

5144

138

138

1458

1458

397

397

2

2

25

25

1811

1811

472

5

467

288

233

55

351

351

183

183

11

11

8

8

6

6

6

55

6

6

32

32

3

3

14

10

4

4

4

4

400

378

15

363

22

22

27

27

27

1839

34

34

100

100

12

12

10

10

39

3

36

22

22

1622

768

10

844

9

9

9

840

31

10

21

5

5

5

5

96

96

355

346

9

39

21

11

7

296

296

13

13

43

43

43

548

548

16

10

97

60

212

153

82

14

14

30

30

17

17

9

9

12
3

9

425

410

410

15

15

94

30

30

64

2

62

101620
52163

4

4

4

17

17

17

24

24

2

22

6

6

6

5

5

5

22

19

19

3

3

17
75

10

10

6

6

4

4

38
18

20

295

136

136

81

81

36

5

31

42

11

31

59

17

13

4

2

2

3

3

22

16

6

15

6

7

2

47

47

47

360
6

2

2

16

16

3

333

18108

62

36

26

2117

2117

1695

34

1578

83

35

35

14199

13

14186

5

5

5

1846

40

40

151

45

12

94

7

7

9

9

122

9

43

12

2

56

240

240

24

24

3

3

21

21

109

81

28

25

25

3

3

5

5

189

4

185

61

7

54

12

12

59

59

199

152

27

15

5

352

139

31

88

94

84

29

55

14

14

43

43

38

2

17

19

36

36

4

4

2

2

334

334

15

146

12

52

7

37

65

65

44

3

3

23

15

3

3

18

12

6

27

5

5

2

2

20

20

31

6

6

3

3

22

16393
3488

666
599

60

7

19

19

39
53

5

9

18

18

26

11

15

11

11

11

87
117

26

4

102

102

217

217

50

26

26

9

9

20

20

182

182

1367
775

73

5

514

55

55

50

23

23

15

15

11

11

58
7

27

24

110

110

780

9

18

40

713

140

4

127

9

15

15

84

84

26

26

168

147

9

12

490
487

3

110
122

12

8

8

30

8

22

31

20

11

57

16

19

22

35

35

32

24

8

17
6

11

70

70

59

39

20

7

7

7

7

8

8

10

10

45

35

3

7

8

8

144

116

4

24

3

3

55

55

47

47

2
270

268

14

14

16

16

207

16

69

8

16

43

34

21

8

8

10

10

19

6

13

11

11

53

53

11

4

7

6

6

46

7

14

14

1319

12

1307

36

36

3

3

5

5

59

59

106

75

75

42

17

25

396

137

206

53

2

2

9

9

13

13

86
77

9

1001

1001

104
167

63

15

9

6

240

240

587

544

23

20

425

425

3

10

3

7

25

9

9

256

256

2

2

21
23

2

4

4

19

19

11

11

36
61

25

11

11

18

18

52

9

9

3

3

17

17

22

22

4

4

11

7

4

9

7

2

30

30

2

19

11

8

83

134

134

21

21

36

11

25

8

8

122

113

9

9
20

11

13
18

5

44

44

25
29

4

29
40

3

8

79

79

5
41

36

78

78

26

3

23

68

68

3

3

7

7

231

231

8

8

8

8

3
98

73

22

81

81

7

2
7

5

79

79

35

44

25
1206

107

2

97

5

3

4

4

3

5

5

17

9

8

2
8

6

44
80

36

4

18

16

16

18

18
3

5

2

8

62
2

60

523

523

26

2

2

42
44

2

9

9

4

4

4

4

2

2

2
134

3

2

18

7

4

98

6
4

2

21

21

46

11

3

27

5

1722

32

6

26

245

245

1445

10

59

5

260

576

114

177

244

6

6

6

11

11

11

54
8375

8023

8023

37

37

209

4

39

166

2

2

50

2

24

24

280

280

280

4

4

4

13
10

3

3

5

5

5

7

7

7

9
5

4

4

6

6

6

937

937

937

632

223

82

412

3

3

3

57

57

57

352

174

174

171

171

7

7

85
45

36

36

8

28

4

4

4

2936
4738

1802

1608

1053

555

194

194

54

7

7

7

47

31

31

16

16

167

3
167

143

2

2

141

141

21

21

21

2

2

2

2

2

6

6

628
37

262

40

40

40

100

100

19

81

22

10

10

10

10

2

2

12

12

12

67

67

65

2

21

21

21

2
7

5

56
6

4
50

46

46

9
266

257

257

257

528

48

28

28

28

28

11

4

4

4

7

7

7

2

2

2

2

7

5

5

5

2

2

2

480

428

85

81

81

2

2

2

2

281

4

4

277

277

62

17

15

2

45

2

32

11

52

52

52

52

59

59

59

59

59

22

37
