# Supplementary material for: Toward a standardized quantitative and qualitative insect monitoring scheme
Source: Ecol Evol. 2020 Apr 2;10(9):4009–20. doi: 10.1002/ece3.6166 (PMC7244892; doi:10.1002/ece3.6166)
Supplement: Supplementary file 7 — Figure S3 [file ECE3-10-4009-s007.html]

Javascript must be enabled to view this page.

magnitude
magnitudeUnassigned

conventional grassland

526830

16

16

16

16

16

5

11

265

52

17

13

13

13

4

4

4

2

2

2

2

33

33

33

33

213

175

95

22

2

20

73

18

2

5

48

80

2

2

78

78

38

38

38

38

3

3

3

3

3

3

526540

2

2

2

2

2

271

271
2

19

19

19

250

242

242

8

8

18
475

5
3

2

311
66

86

68

47

21

18

18

18

8

8

6

6

4

4

40

40

40

47

34

34

13

13

3

3

3

51

51

49

2

6

6

6

6

135

135
10

21

21

104

104

525783

14925
3507

26

26

5266
639

348

348

156

156

254

93

161

24

24

5

5

338
506

168

15

15

104

104

45
19

26

12

12

158

158

1956
650

1292

14

99
104

5

900

900

21

21

19

19

37

37

17

17

17

362

186

186

176

174

2

304

288

40

248

16

16

3958

7

7

2

2

3949

3949

2

2

2

11

11

11

34
6

28

16

12

28

28

28

333

333

4

329

6

6

6

83

83
4

79

26

26

26

10

10

10

2

2

2

275
67

6

6

35

35

3

3

11
78

67

31

31

50

5

5

82
59

23

55
317

2

2

59

59

25

25

125

125

2

2

16

16

4

4

6

6

23

4

13

6

239

4

4

3

3

232

232

318

318

318

147

75

96

2
51

49

49

43

6

2

2

2

2

39074
1991

3

3

3

22313

1159

1159

9381

3246

6135

4982

4982

455

455

1175

1175

863

863

211

211

5

5

20

20

904

904

144

144

2987
11

410

2566

27

27

26

26

26

25

25

25

3779

29

29

392

260

132

1066

1066

22

22

204

204

9

9

97

77

4

16

54

54

97

97

8

8

103

103

37

37

57

57

711

33

53

625

712

363

349

181

181

22

22

11

11

172

172

172

2

2

2

196

196

196

297

91

9

4

20

7

51

26

26

5

3

2

175

175

14

14

14

1571

38

38

1533

1533

498

498

498

13

13

13

2512

13

13

2392

2333

59

107

107

54

30

30

24

24

59

59

30

13

9

7

220

7

7

53

53

8

8

116

116

10

10

4

4

22

22

1622

1622

1622

474

13

13

12

12

51

51

44

44

7

7

5

5

161

2

152

7

49

49

132

132

433

433

45

388

7

7

7

2110
206

32

32

69

69

310

310

909

625

284

301

301

283

283

37

27

27

10

10

3

3

3

46

46

26

20

3

3

3

292

30

10

20

26

26

45

45

8

8

49

49

8

8

3

3

83

83

40

18

22

118

118

118

162

159

159

3

3

9602
10958

1356

1132

994

138

224

224

124
23075

73

9

7

2

26

26

38

38

11

11

11

2

2

2

1416

121

115

6

72

72

115

115

83

83

1025

1025

44

9

9

35

35

203

203

203

118

20

20

46

27

19

43

43

9

2

7

3

3

3

26

26

26

1184

7

7

1177

1177

161

161

161

342

172

172

170

170

809
12

72

72

4

4

53

53

28

28

5

5

8

8

262

262

67

19

45

3

298

7

18

4

32

237

2

2

2

4

2

2

2

2

2888

2888

2439

418

31

5

5

5

213

15

15

64

64

11

11

15

15

106

106

2

2

2

2

2

288

288

288

221

12

12

209

209

1995
34

41

41

1389

1389

5

5

255

255

159

159

112

112

253

249

249

4

4

333

72

72

242

242

19

19

37

37

37

8

8

6

2

12310

7

7

9049

2291

4475

59

2224

3254

88

3095

71

83101
37774

2722

252

17

230

5

74

74

65

2

63

275

246

16

4

9

16

16

306

32

274

249

235

2

10

2

2

2

4

4

144

144

10

10

571

270

13

26

262

276

36

237

3

18

18

25

25

267

267

3

3

46

46

10

10

25

14

11

84

82

2

936
17

85

85

2
6

4

56
51

5

98
18

14

48

18

6

324

324

6

6

9

64
5

48

2

9

2

2

17

4

4

39

8

31

2
34

32

15

15

11

11

2

2

10

10

131

3

3

117

8

451

372

372

70

70

9

9

1016

5

5

876

38

451

25

19

343

135

135

33

33

6

27

2
7

5

5

128

2
128

3

123

132

27

27

49

2

47

43

8

2

3

9

6

15

13

4

3

6

197

51

51

18

18

128

128

2

2

2

245

54

3

51

134

122

12

10

10

40

2

12

12

7

7

7

7

47

47

47

355

355

175

13

100

40

27

9

2

2

7

7

24

24

24

14

14
12

2

357

17

2

7

8

340

174

174

16

3

155

6

175

10

10

165

165

1303
255

745

6

682

57

2

2

28

28

258

6

96

156

15

15

24
2

22

22

8
34

20
13

7

2

2

2

2

2

2

2

2

2

222

222

222

2

2

27435

5884

5884

545

8

453

84

1123

1123

15261

15254

7

4622

4622

4

9250
894

18

18

244
134

81

29

3

3

61

61

22

22

12

12

21

21

538

4

4

3

3

6

6

3

3

112

112

438

438

9

45

3

42

211

211

2

2

20

20

486

209

34

243

73

3

3

2

2

14

75
25

5

45

39

7

32

102

102

3

2

2

13

13

17

17

23

17

17

57

57

230

9

139

131

8

172

172

4

4

15

2

2

35

12

23

70

70

775

226

381

168

153

141

12

6

4

2

45

45

66

20

20

47

47

52

101

20
30

2

8

72

72

112

112

26

26

21

21

50

50

25

25

45
53

8

3

3

2

2

37

37

7

7

2

2

491

491

40

40

19
31

9

3

3

3

48

48

14

14

46

4

42

1895
685

1189

21

184

63
26

37

14

14

11

11

8

13

5

8

10

10

16

29

29

57

57

204

204

2

2

2

2

39

39

32

7

18

2

5

25

15

15

15

4

4

4

7
5029

25

10

10

15

15

5

5

5

4992

4939

76

4

5

155

4699

53

53

110

110

51

51

40

40

19

19

38

38

38

38

1021

23

23

10

13

158

2

2

156

156

840

29

29

328

328

483

483

90
9

15

15

15

63

63

9

15

39

3

3

3

2
37

35

35

30

5

94350
347954

111

111

111

512

69

69

12

12

43

43

301

9

5

4

73

73

5

5

20
721

2

2

3

3

4

4

18

18

523

18

483

16

6

31

31

4

4

107

82

25

9

9

41566

7056
525

90

2351

229

7

18

18

20

2921

64

181

623

9

417

409

8

173

82

91

42

42

51

48

3

2486

41

566

69

865

11

4

930

297

48

249

209

209

72

72

18031

6

158

517

15640

207

1503

131

12592

11911

75

606

9

9

421

12

12

409

409

400

400

400

479

6

6

473

473

38946

5444

136

5308

320

320

334

266

19

49

595

595

15
8437

282

7353

765

22

12187

11512

391

2

62

197

23

196
1713

34

1211

272

24

24

1427

851

175

87

45

96

173

39

39

9

4525

35

4299

191

2247

2247

204

204

79

17

11

51

1362

1362

10

10

10

5711

414

5

57

57

5227

807

33

879

3

81

755

1273

1112

284

8

8

2369
4

2

5

5

72
299

41

11

22

44

109

67

67

711

711

682

682

8

8

446

38

327

81

11

11

47

47

87

87

1411

1411

117

375

919

36429

37

37

31455

8536

767

839

4426

9065

10

7812

692

692

405

405

10
3840

345

45

2640

464

336

578
13

7

7

3

3

73

73

36
10

2

6

5

13

17

17

263

147

53

59

2

2

100

32

13

13

36

3

3

27

12

4

3

8

24

24

13

13

2

2

226

221

221

5

5

970

480

208

272

490

257

188

45

13900
59

76

76

69

69

20

20

31

16

15

991

991

58

58

5

5

577

577

1767

1767

10
1726

1716

5

5

282

282

324

324

63

63

75

75

66

66

63

6

57

345

345

697

8

650

35

4

6601
85

6516

56935

2

2

12

2098

2098

171
54823

31

38

2536

14271

1258

6458

20191

8390

1211

67

196

5

942
45

90

90

387

387

89

24

65

321

321

10

10

56
710

28

2

2

5

74

74

24

9

15

30

3

12

15

5

2

2

24

9

9

26

94

4

2

29

43

6

11

4

116

116

53

3

3

2

32

2283

2267
235

4

20

32

145

7

294

11

52

135

224

33

416

8

77

2

572

16

16

3369

326

155

171

1009

1009

720

669

51

1310

1310

4

4

19

5

5

5

5

9

9

14

8

8

6

6

12383

445

445

2424

2424

57

57

2163

1237

652

103

146

25

2237

414

322

1501

4400
5057

163

14

76

19

385

49

49

49

8

8

8

109

4

4

10

22
91

19

17

2

18

9

4

5

5

5

4
438

2

2

59

59

25

25

12

8

2

6

47

45

2

12

254

7

2

5

92

2

146

2

2

6

7

11

11

11

44

2

2

16

16

26

1311

844

59

7

778

7

7

26

26

225

225

133
107

26

76

46

30

8
302

51

51

93

93

28

24

4

122

10

112

4969
700

2

2

107

25

25

233
12

221

2

5

27

14

55

47

2

33

12

15

12

8

9

20

124
2996

64

596

17

14

42

5

8

32

12

38

17

26

24

11

155

13

7

189

230

29

247

37

28

33

4

4

4

13

2

4

4

13

34

173

699

37

7

461

270

191

2

2

53
205

152

22

17

5

8

8

8

38

38

8
75

5

5

62

3489
184

110

110

3195

3195

395

170

104

104

121

9

63

40

9

395
40

82

82

2

4

4

45

7

7

186

186

29

11

18

131

131

131

3

3

3

2

2

2

476

22

22

2

2

9

9

2

2

14

14

27

4

19

4

181

181

44

44

98

98

4

4

38

34

4

35

35

1763

36

26

10

3

3

146

146

28

28

89

4

85

42

3

2

37

142

3

9

4

126

22

14

8

11

11

567

567

17

15

3

12

2

2

629

5

13

10

172

13

185

4

59

5

63

41

14

16

9

20

14

14

132

20
65

8

37

67

16

51

18
22

4

4

516

341
280

54

7

150
175

25

14271

448

361

87

159

159

2

2

182

182

543

543

89

89

2

1621

1621

2509

2509

64

64

123

123

3

3

19

19

509

509

354

354

116

116

28

26

2

14

14

14

14

17

17

39

39

285

285

1954

1290

31

633

75

75

8

8

79

79

119

119

737

737

152

152

51

51

390

387

3

647

647

300

300

95

8

77

10

177

177

85

85

108

108

2154

2154

3014

3014

3014

2

2

2

36

36

175

42

37

5

36

36

2

4

91

91

9

3

6

6

6

6

6

6

2

2

2

2

4

4

4

4
