# Supplementary material for: Toward a standardized quantitative and qualitative insect monitoring scheme
Source: Ecol Evol. 2020 Apr 2;10(9):4009–20. doi: 10.1002/ece3.6166 (PMC7244892; doi:10.1002/ece3.6166)
Supplement: Supplementary file 8 — Figure S4 [file ECE3-10-4009-s008.html]

Javascript must be enabled to view this page.

magnitude
magnitudeUnassigned

conventional forest fringe

441295

134

134

134

134

134

7

127

2

2

2

2

2

2

440996

123
440154

37

37

37

7

30

556

556

63

63

372

372

121

121

1558
25211

383

383

383

19

19

19

35

35

21

14

223

223

223

209

207

207

2

2

6

6

6

383

383
191

192

4

4

4

13936
814

14

14

30

30

43

43

17

17

19

19

136
408

272

479

479

93

93

125

125

48

48

37
23

14

2

2

11465
9624

1841

133

83
95

12

10

10

26

26

7

7

11

11

54

54

6

2

2

2

1500

1494

268

1226

6

6

3

3

3

412
61

5

5

3

3

3

3

169

46

114

9

95

95

37

37

4

4

26

26

9

9

225

89

89

136

8

128

4

4

4

3
18

15

122

7

7

115

115

65
313

32

32

122

14

108

2

2

19

19

69

69

4

4

5853

172

172

18

18

5589

5589

74

74

54625
448

5

5

5

166
5700

10

10

220

220

388

325

41

22

119

119

689

31

658

378

378

2974

2974

214

214

542

283

259

24

22

22

2

2

180

180

180

24

24

24

597

108

108

11

11

6

6

66

66

54

54

345

345

7

7

681

681

681

19010

1667

1667

5021

5021

2474

88

7

2379

62

62

1454

1454

2021

2021

5

5

14

14

3292

3200

92

42

42

125

125

43

43

6

6

2695

2695

62

62

27

27

13569

13569

13569

34

20

20

14

14

195

16

16

10

10

4

4

75

75

4

4

24

24

17

17

45

26

19

71

71

71

33

33

33

2993

231

44

187

58

58

65

65

602

592

10

1213

1213

26

26

4

4

6

6

69

69

18

18

55

55

366

366

280

280

363

363

88

90

185

8

6

6

2

2

17

17

4

13

5846

1775

767

1008

23

23

42

5

16

21

78

78

234

27

207

390

390

869

589

184

96

122

9

107

6

939

939

391

391

6

6

435

435

4

4

30

30

454

454

31

31

23

23

239

239

239

13

13

13

71

5

5

58

8

4

11

25

10

8

2

6

36

36

36

152

152

150

2

164

157

157

7

7

20

20

20

1527

106

106

1399

147

1252

22

2

3

17

23

23

23

87

87

87

24

24

24

165

165

165

1736

1736

1736

92

92

24

68

21

21

21

46

23

23

23

23

112

112

112

266

266

266

33

33

33

287

256

256

256

31

31

31

336
16571

1025

847

8

839

149

147

2

23

23

6

6

6

6

6

3130
18

2939

2838

101

14

14

5

5

44

44

16

16

72

72

3

3

19

19

45

40

40

5

5

20

20

20

46

2

2

44

44

786

786

786

26

26

26

24

17

17

7

7

177

177

177

152

7

7

14

9

5

74

5

69

57

21

36

546

546

546

82

71

71

11

11

2

2

2

72

72

72

35

35

2

7

17

7

2

9

3

3

2

2

4

4

12

12

12

94

3

3

82

82

9

9

418

8

8

35

35

22

22

26

26

17

6

11

2

2

21

21

2

2

18

18

48

48

56

28

28

163

16

6

42

9

16

2

72

18

18

18

6557

2381

35

164

2166

16

4075

9

389

14

15

3648

5

5

87

87

9

9

27

27

27

1367

76

76

12

12

601

601

446

446

208

208

24

24

563

493

493

2

2

58

58

2

2

8

8

18

18

18

969

969

969

9

9

9

801

801

801

166

114

521

63267
22231

891
14354

9

9

6

6

26

12

14

8

8

19

19

51

28

15

15

7
3

4

18

5

13

167
155

10

2

5

3

2

8

8

30

30

18

4

9

5

413

135

7

35

236

82

34

21

13

14

16
45

29

63

63

8
10

2

41

41

1116

1116

217

535
655

57

63

166

166

93

78

3

12

21

21

52

52

2

6

6

12

12

14

58
77

9

10

4

4

55

55

37

23

11

3

38

38

5

192

1047

28

53

51

513

100

302

25
58

3

30

6

100

100

27

27

180

180

405

349

33

23

3

3

4

4

6

6

273
6

267

24

24

136

103

33

107

55

52

341
1020

632

47

441

345

94

2

169

169

70

70

5

5

24

24

3

3

6

6

13

13

4

4

99
96

3

8

8

270

241

29

13
2

11

4

66

66

79

79

275

254

21

20

20

27

27

141

141

3

3

73

6

67

51

31

9

11

2728

11

11

82

82

2

2

7
23

6

10

56

56

129

129

11

11

37

74

8

66

30
26

4

34

34

38

38

3

3

593

593

19

2

17

22
206

106

78

19
17

2

9
3

6

201

80

64

57

67
62

5

4
10

4

4

2

14
12

2

2

3

3

3

5

5

5

339

339

339

22
81

17
25

8

22

22

3

3

2

2

2

2

5

5

64

31

4

27

33

25

3

5

4

4

4

13

13

13

3

79
972

2

2

20

20

3

3

33

3

2

28

30
11

19

17

3

14

5
170

12

20

34

99

3

3

27

4

4

37
5

19

6

2

5

6

2
145

21

3

15

36

60

8

17

2

2

47

3

16

6

22

2

73

4

2

67

10
42

15

17

8

8

85

85

52

52

52
7

45

7

7

7

7

23
4

19

19

2

284

118

118

94

7

87

59

59

7

5

2

6

6

83

83

83

845

845

306

539

122
6

15

15

66

66

2

2

33

33

4935

82

48

34

1049

1049

626

626

3178

609

1080

14

32

118

9

809

507

2

8

8

8

2

2

2

29
21

2

2

6

6

94

69

7

42

6

14

25

25

41
652

36

36

36

36

102

94

2

6

57

57

378

2

96

60

220

2

2

3
13

10

10

3

3

3

6
393

347

18

5

2

3

17

17

14606

135

135

1374

1374

5955

5955

7142

7108

34

2

2

2

1274

1274

35

43

10

1003

20

2

114

29

13

5

1802

39

39

579

3

576

7

7

42

30

4

8

88

88

41

21

20

18

18

17

17

3

3

266

3

15

248

110

110

154

32

23

91

8

6

6

89

2

87

268

176

92

75

28

4

38

5

6505
9767

3262

1920

1920

1342

785

557

270

31

31

26

5

189

189

189

50

50

50

57646
267373

31

31

31

5

3

3

2

7

7

285

285

285

11234
2110

4
125

93

28

3

62

47

9

6

61

3

2

5

5

187

2

2

16

562

20

528

14

6

14

61

13

5

8

9

9

9

6378
485

33

1138

7

97

161

25

20

3

2

35

1431

12

22

158

32

2

228

3

20

7

41

35

10

127

50

34

104

99

124

65

48

8

4

87

19

516

19

50

210

146

185

152

7

317

41

56

38

18

8

39
710

671

52

3

734

2

640

640

640

9

9

9

5489

1100

38

858

204

4389

7

1803

1129

1450

55

55

55

7
223

29

2

17

2

8

187

12

3

2

29

101

27

13

735
164

3

3

2

2

9

9

12

12

11

11

3

410

4

4

156

4

51

191

6

45

12

12

16

16

18

18

24

24

2646
66

40

40

3

3

12

6

3

85

4

5

14

8

4

6

12

310

233

233

3

7

57

7

3

24

10

9

7

7

100

100

34

2

30

15

55

55

4

4

10

10

531

16

4

12

4

4

230

25

10

3

14

31

342

82

61

19

2

54

35

3

15

26

21

9168
533

5995

5835

160

294
2300

85

1871

3

47

340

340

880

31

31

528

321

38

107

122

54

13539
3

17

397
17

12

19

349

772

35

514

11

42

170

7

3

4

555

21

534

51

37

14

511

511

3974

6

167

3596

205

3347

3334

13

29

12

17

16

13

3

37

37

1185

6

1153

26

134

134

2
2504

172

599

52

1679

9456

100

4

96

56

56

3

3

936

936

746

746

134

134

27

27

43

43

38

38

29

3

3

101

101

7

7

546

546

1351

1351

17

17

938

938

63

63

52

52

11

11

2

2

14

14

16

16

3

3

64

64

46

46

200

200

22

22

205

205

6

6

404

404

1672
15

1657

32

32

788

788

23

23

5

5

62

62

6

6

144

137

7

203

169

34

338

206

132

1059

1059

1059

124
103

11

10

14855
752

7

2

5

301

16

15

270

77

77

886

886

28

28

1053
2

1051

16
126

110

174

174

84

84

71

71

149

97

52

875

706

169

1363

682

681

3406
55

3351

18

18

333

333

14

14

376

376

298

298

4464

2

4462

8
3341

94

94

262

9

89

2

162

780

780

49

45

4

1176

1176

16

2

14

227

227

81

81

6

6

71
642

36

20

360

31

38

70

2

12

2

197

24

14

10

3

3

3

3

8

2

6

15

15

36

36

6

6

46

46

51

51

5

5

20

20

1136

1136

1136

53

53

53

3506

207

207

22

22

63

63

9

9

276

113

25

125

13

3
142

103

36

22

20

2

1726

65

15

17

2

6

793

57

16

17

233

32

222

16

15

5

68

147

13

3

10

149
37

4

14

19

4

4

52

15

56

10

30

5

5

6

89

9

9

154

6

3

34

4

4

103

24

24

433

433

33

31

2

79

79

35

8

8

25

17

8

2

730

35

17

18

2

22

664
245

39

18

45

166

48

33

70

7

35414

402

402

32154
23

19

23

224

3307

102

75

20392

55

45

1125

6731

33

2766

2766

56

56

36

36

1025

4

4

3

3

14

2

12

27

9

18

38

33

5

30

30

58

58

14

14

74

74

17

17

2
157

6

37

104

8

24

15

2

7

565
73

53

79

18

14

4

106

218

80

12

3

3

65

36

29

3715

128

140

140

6
3447

1170

2271

14
28

14

44

44

44

763
31

17

17

73
22

5

37

2

4

3

166

35

8

23

100

2
350

76

270

2

14

14

42

5

2

3

7

25

3

3

31

22

2

4

3

36

21

15

3705

3705

8

1354

2343

7

7

7

268

266

266

2

2

613

115

73

73

201

22

125

54

224

37

174

13

5649

199

130

3

66

4549

95

117

416

20

445

13

593

410

2440

25

25

834

42

20

20

2

59

6

6

44

9

35

9

9

4208

11
4208

2

1111

123

39

5

2

1032

17

91

4

120

4

7

13

791

48

62

726

21883

95

95

11276

58

11218

18

18

14

14

1081

782

203

16

4

76

1275

747

526

2

3077
4221

70

2

57

980

19

16

3903

3903

694

95
682

260

327

12

12

35428

26

26

6

6

3745

2902

40

794

9

70

24835
10

1960

21746

593

4

2

471

49

24

24

1031

5

577

449

14

14

4

18

7

11

4014

11

60

2154

1382

126

234

28

4

15

1278

69

1055

154

167

160

7

129

126

3

67

67

1338

6

4

2

885

885

307

10

10

120

120

10

8

2

457

2

2

12

12

54
6

48

98

34

64

261

244

5

12

30

8752

871

871

4234

38

7

2766

235

1173

15

240

240

5

5

3402
15

80

3113

61

133

806

806

806

13

13

13

63

63

63

49

49

23

12

14

66

57

53

4

9

9

2773

173

173

17

17

191

175

16

2392

2392

147
1317

15

15

30

9

21

3

3

13

13

2

2

31

96

6

67

23

27

953

953

250

50

50

200

131

30

39

32

23

23

7

7

2

32
770

28

28

11

11

407

407

155

155

66

66

71

71

329
24

60

60

60

231

231
7

224

6

6

6

8

8

8

2

2

2

2

3
935

808

805

2

18

129

10

646

3

3

67

64

64

3

57

57

57

34

34

203

28

28

28

175

165

35

35

106

106

24

24

10

10

10

605
2

2

2

429

183

183

183

76

76

65

9

2

16

16

16

17

17

17

27

27

27

105

105

57

48

5

5

5

167

167

7

7

160

160

2

2

2

2

3

3

9

9

3

3

3

3

6

6

6

6

154

37

23

23

23

23

8

8

8

8

6

2

2

2

4

4

4

117

112

49

49

49

63

54

19

33

2

9

3

6

5

5

5

5
