# Supplementary material for: Toward a standardized quantitative and qualitative insect monitoring scheme
Source: Ecol Evol. 2020 Apr 2;10(9):4009–20. doi: 10.1002/ece3.6166 (PMC7244892; doi:10.1002/ece3.6166)
Supplement: Supplementary file 9 — Supplementary Material [file ECE3-10-4009-s009.docx]

**Supplementary Information**

Supplementary Table S1: Wet and dry biomass of the 44 Malaise trap samples from April 10 to October 19 2018 on the study sites on the organic (M1 – M2) and on the conventional farmland (M3 – M4). Wet biomass was measured following the recommendations of Ssymanck et al (2018).

Supplementary Table S2: Arthropod and Euarthropod BINs and species names resulting from High Throughput Sequencing (BLASTed against BOLD and GenBank) of the 44 Malaise trap samples from April 10 to October 19 2018 on the study sites on the organic (M1 – M2; pp. 2-57 in S2) and on the conventional farmland (M3 – M4; pp. 58-113 in S2). Note: The list does not include the conventionally identified macrolepidopterans which were sorted out prior to DNA extraction.

Supplementary Table S3: 412 Lepidoptera species collected in the 44 Malaise trap samples from April 10 to October 19 2018 on the study sites on the organic (M1 – M2) and on the conventional farmland (M3 – M4). Macrolepidoptera were sorted out of the samples before homogenization and DNA extraction and identified in a conventional, morphology-based approach. In addition, data from High Throughput Sequencing (HTS) are added (see Supplementary Table S2). * not collected at light; ^(1)^ according to current taxonomy belonging to Microlepidoptera. Red Book Data: 1 = critically endangered; 2 = endangered; 3 = vulnerable; V = near threatened; G = threat assumed but data deficient; R = extremely rare and local species.

Supplementary Table S4: 371 Lepidoptera species and numbers of collected individuals at light from April 10 to October 19 2018 on the study sites on the organic and on the conventional farmland, identified in a conventional, morphology-based approach. Red Book Data: 3 = vulnerable; V = near threatened.

Supplementary Figure S1: Interactive KRONA files (.zip) for COI BLAST matches after High Throughput Sequencing of arthropods collected in 2018 in the Malaise trap M1 (organic farm, open grassland), M2 (organic farm, forest fringe), M3 (conventional farmland, open grassland) and M4 (conventional farmland, forest fringe).
